# Determinants of Parental Satisfaction with Nursing Care in Paediatric Wards—A Preliminary Report

**DOI:** 10.3390/ijerph16101774

**Published:** 2019-05-20

**Authors:** Agnieszka Kruszecka-Krówka, Ewa Smoleń, Grażyna Cepuch, Krystyna Piskorz-Ogórek, Mieczysława Perek, Agnieszka Gniadek

**Affiliations:** 1 Department of Clinical Nursing, Institute of Nursing and Midwifery, Faculty of Health Sciences, Jagiellonian University Medical College, 31-501 Kraków, Poland; agnieszka.kruszecka-krowka@uj.edu.pl (A.K.-K.); grazyna.cepuch@uj.edu.pl (G.C.); mieczyslawa.perek@uj.edu.pl (M.P.); 2 Chair and Department of Management in Nursing, Faculty of Health Sciences, Medical University of Lublin, 20-059 Lublin, Poland; ewasmolen@op.pl; 3 Nursing Department Faculty of Health Sciences, University of Warmia and Mazury, 10-719 Olsztyn, Poland; piskorz@wssd.olsztyn.pl; 4 Department of Nursing Management and Epidemiological Nursing, Faculty of Health Sciences, Jagiellonian University Medical College, 30-501 Kraków, Poland

**Keywords:** satisfaction with care, nursing care, child, parents

## Abstract

*Background*: The quality of medical services for a child and their parents are tantamount to a sense of satisfaction with care. Purpose: The assessment of determinants of parental satisfaction with nursing care in paediatric wards. *Methods*: The study covered 336 parents of children hospitalised in paediatric departments and was based on the “EMPHATIC” questionnaire, standardized and adapted to Polish conditions. *Results*: The mean score of the overall parental satisfaction was high, amounting to 4.19 points. The lower level of satisfaction with nursing care was reported in parents of children under the age of 6 years, admitted in an emergency mode with a diagnosed post-trauma condition and those with higher education. The duration of hospitalisation, sex and age of parents did not have an influence on the satisfaction with care. *Conclusions*: The age of the child, admission mode and education of respondents are determinants of parental satisfaction with nursing care.

## 1. Introduction

One of the most important goals of modern healthcare management systems is to provide high quality medical services [1]. The functioning of a modern paediatric hospital requires a comprehensive approach to the child’s health problems, developmental issues and family situation [2,3]. For the patient, the quality of medical services is tantamount to a subjective sense of satisfaction with care [1,4]. Satisfaction with medical care, including nursing care, is a multidimensional concept which is defined in various ways and depends on the degree of correspondence between meeting expectations of the patient and his/her family and the perception of care they are provided with [5]. It is also an important indicator of satisfaction with the comprehensive hospital care [6,7]. In case of a child, the satisfaction with care is also assessed by parents or guardians [8], who have the right to be present, participate and co-decide medical care issues [9]. A sense of parental satisfaction with care is determined by their individual needs, experiences of previous hospitalisations and changing external factors, including systemic factors [5,6,7,8,10,11]. Parents’ satisfaction with care may also be influenced by other factors such as child’s health condition and its impact on family functioning, child’s emotional condition, clinical stage of the disease and maintaining continuity of care from hospital admission to discharge including preparing parents for continuing nursing care in home environment [12,13,14,15,16]. The assessment of parental satisfaction is an important part of the holistic nursing care for a paediatric patient which allows finding parents’ attitude towards the services they are provided with [5,7,10], and towards respecting patients’ rights during hospitalisation. In such a context the assessment of satisfaction with nursing care has also an important control function and allows for protecting patients and their families from latrogeny [17,18]. The opinions of beneficiaries are also an invaluable source of information allowing for analysing the situation and introducing changes in order to optimise the activities of medical providers according to documented presumptions [12,17,19]. Abilities of the nursing staff to meet patients’ expectations contribute to an increase in the competitiveness of a health care unit and patients’ positive opinions help to advertise it [20,21]. It can be assumed that a satisfied patient is more likely to follow medical and nursing recommendations and regularly take part in a medical check-up [22,23,24]. Moreover, patient’s high satisfaction from nursing care may determine the satisfaction of the whole nursing team and their work and play an important motivating role [25].

In Poland, the evaluation of satisfaction of parents/guardians with childcare in paediatric departments is a relatively new research area; at the same time, it is one of the priority recommendations for healthcare providers. National research in this area has been so far conducted mainly using non-standardized original questionnaires which makes it impossible to draw objective conclusions and to compare results [8,10]. In Poland, the first study, which was based on the standardized “EMPHATIC” questionnaire developed by Latour et al. [26,27,28,29,30], was carried out in the years 2012–2014 [10,31].

Given the above, there is a need for further research using standardized tools for the assessment of parental satisfaction with nursing care in paediatric departments. The analysis of scientific reports suggests that the implementation of the evaluation of satisfaction with medical care, including nursing care in health facilities should be a priority, because this would allow for finding determinants and optimizing the activities focused on the needs of the child and its family.

Study objective: The assessment of determinants of parental satisfaction with nursing care in paediatric wards.

## 2. Materials and Methods

### 2.1. Trial Design and Participants

The study was carried out in the years 2016–2017 in general-paediatric and specialist (non-surgical) departments at the University Children’s Hospital and the Provincial Specialist Children’s Hospital in Krakow. The study was approved by the directors of the facilities and parents. The study was conducted in a group of 336 parents of children hospitalised in general-paediatric and specialist departments (non-surgical) on the day of discharge from the hospital. After receiving the medical information letter, the parent (being the main caregiver during hospitalisation) completed the questionnaire.

The inclusion criteria were as follows: Permanent or periodic, not shorter than 12 h a day presence of a parent with a child during hospitalisation, hospital stays lasting more than three days, and voluntary consent to participate in the study.

The exclusion criteria included: Unwillingness to participate in the study, hospitalisation lasting less than three days, the absence of parents during hospitalisation or their occasional presence (shorter than 12 h a day), or a terminal period of the child’s life.

Parental participation in the study was voluntary and anonymous. Each parent was provided with the full information about the purpose of the study and the possibility of resigning from the participation at any stage without giving a reason.

### 2.2. Outcome Measures

The study was based on the method of diagnostic survey using a questionnaire technique including:

1. A standardized questionnaire for the evaluation of the level of satisfaction of parents/guardians with nursing care—“EMPHATIC” questionnaire developed by Latour et al. [30], adapted to the Polish conditions by Smoleń and Ksykiewicz-Dorota [31].

The tool uses five major criteria evaluating satisfaction with nursing care for a child and specific criteria: criterion I “Information”—contains eight specific criteria, criterion II “Care and treatment”—contains nine specific criteria, criterion III “Availability”—contains two specific criteria, criterion IV “Parental participation”—contains six specific criteria, criterion V “Professionalism/Professional approach”—contains 14 specific criteria.

Each specific criterion was assessed by parents in a five-point Likert scale from 1 to 5 points, where “1” means “I am very dissatisfied”, and “5”—“I am very satisfied”. The evaluation of satisfaction was presented using point values, with the accuracy of two decimal places. The assessment score in the range from 4 to 5 is considered as satisfaction with care.

Due to the preliminary character of the study, only the material referring to the main criteria, which was an average value of the scores obtained in detailed criteria, were taken into account in a statistical analysis. No statistical analysis for particular detailed criteria was carried out. What was presented were only the results showing which detailed criteria received from respondents that had the highest and lowest scores.

2. The summary of socio-demographic data: Age of the child, reason for hospitalisation, duration of the child’s stay in the ward, the mode of admitting to the hospital, parental age, sex and education.

A division of children into age groups was created in accordance with the paediatric classification corresponding to the developmental stages [32], while the age of parents was assigned to one of three categories: below 30 years of age, 30–40 years of age, above 40 years of age. The questionnaire included an emergency or elective mode of admission to the hospital.

### 2.3. Statistical Analysis

The statistical analyses were performed using IBM SPSS Software for Windows, Version 23.0 (IBM Corp, Armonk, NY, USA). The elements of descriptive statistics and the following tests were used: V-Cramér, Phi, Tb—Kendall, Tc—Kendall, U Mann-Whitney and Kruskal-Wallis. Threshold of statistical significance for all tests was set at *p* < 0.05.

### 2.4. Ethics

The study was carried out according to the ethical principles of the Helsinki Declaration. The protocol of the study was approved by the Bioethical Committee of the Jagiellonian University (No. 122.6120.254.2016). Participation in the study was voluntary and written informed consent to participate in the study was sought from all the study subjects.

## 3. Results

### 3.1. Participants

The study group consisted of 336 parents. Women accounted for 84.5% (*n* = 284) of the respondents. The mean age of parents was 33.57 (Min 19, Max 59). In specialist departments, 63.1% of children (*n* = 212) were hospitalised and 36.9% (*n* = 124) stayed in general-paediatric wards. The mean duration of hospitalisation was 10.64 days (Min 3, Max 119). The emergency mode of admission to the hospital concerned 66.7% of children (*n* = 224). The details are presented in Table 1.

### 3.2. The Assessment of Parental Satisfaction with Nursing Care

The mean score of overall parental satisfaction with nursing care was 4.19 points. The results of parental evaluation of satisfaction with nursing care were high for the major criteria I–V.

The highest mean score (4.38 points) was obtained for major criterion III “Availability”, while the lowest score (4.11 points) was achieved for major criterion II “Care and treatment” and IV “Parental participation”.

The assessment of parental satisfaction with nursing care, taking into account specific criteria, showed that for the major criterion V “Professionalism/Professional approach” the highest mean score—4.57 points—was reported for the specific criterion “The nurse provided high-quality care regardless of race, religion, sex and education”.

High scores of the parental assessment were also given for the specific criteria: “The immediate environment of the child was clean” (4.56 points), “The hours of visits at the ward were flexible” (4.52 points) and “The child’s health was the highest priority for the nurse” (4.47 points). The lowest mean score was obtained for the specific criterion “The nurse introduced herself by name and surname”—2.67 points.

A lower mean score in the assessment of parental satisfaction with care was also achieved for major criterion IV “Parental participation” for the specific criterion “The nurse asked about the expectations of parents/guardians regarding childcare” (3.48 points). The low level of parental satisfaction with care also involved major criterion II “Care and treatment” with respect to providing mental support by a nurse (3.77 points), preparation for care after discharge from the hospital (3.77 points) or emotional support (3.92 points); and the major criterion I “Information” with respect to informing about the course of nursing care on a daily basis (3.78 points), too.

### 3.3. Parental Satisfaction from Nursing Care and the Reference Level of Polish Hospitals and Their Wards

All main criteria as well as general satisfaction from nursing care were evaluated higher by parents of patients hospitalised in Provincial Hospital than of those hospitalised in University Hospital, however only for the first main criterion “I Information” the differences were statistically significant (*p* = 0.005).

No statistically significant differences between the assessment of particular criteria of satisfaction with nursing were found depending on the ward type—general-paediatric vs. specialist departments (*p* > 0.05).

### 3.4. Parental Satisfaction with Care vs. the Age of Hospitalised Children

Taking into account the division of children into age groups used in paediatrics, it was shown that compared to parents of children from other age groups, parents of children aged 6 to 12 years better evaluated both the overall satisfaction with nursing care (*p* = 0.04) and the level of satisfaction within the major criterion “I. Information” (*p* = 0.02), “II. Care and treatment” (*p* = 0.04) and “V. Professionalism/Professional approach” (*p* = 0.03).

However, the division of children into only two age groups - up to the age of 6 years (younger children) and above the age of 6 years (older children) statistically significant differences were demonstrated both in terms of the overall satisfaction assessment and the evaluation of all major criteria, with the exception of criterion “III. Availability” (Table 2).

### 3.5. Parental Satisfaction with Care vs. Admission Mode to the Hospital, Type of Clinical Diagnosis and Duration of Hospitalisation

Admission to the hospital in an emergency mode was statistically significant, and more often involved children up to the age of 6 years (*p* < 0.001), while older children, i.e., over 6 years of age, were more frequently admitted in an elective mode (*p* < 0.001).

Emergency admission of children to hospital was statistically more common in University Hospital than in Provincial Hospital (*p* = 0.001, Phi = 0.19, Chi^2^ = 11.74, df = 1).

Emergency hospital admission was significantly more common for children suffering from an acute disease, after an injury or poisoning (Chi^2^ = 107.63, df = 5, *p* < 0.001, V Kramer = 0.57).

Parents of children admitted to the hospital in an emergency mode was statistically significant, with lower evaluated nursing care within the major criteria: I “Information”, II “Care and Treatment”, IV “Parental participation” and general satisfaction with care (Table 3).

Parents of children diagnosed with post-trauma conditions reported a lower satisfaction level within the main criterion I “Information” than parents of children with another type of clinical diagnosis (*p* = 0.011).

The duration of the child’s stay in the hospital did not have an influence on the overall assessment of parental satisfaction with nursing care (*p* = 0.48) and the evaluation within the major criteria: I “Information”, II “Care and Treatment”, III “Availability”, IV “Parental participation” and V “Professionalism/Professional approach” (*p* > 0.05). The lowest satisfaction from nursing care in all main categories was observed in the case of parents of children whose hospitalisation lasted between 8 and 14 days, however, this difference was not statistically significant.

### 3.6. Satisfaction with Care vs. Parental Sex, Age and the Level of Education

There was no relationship between parental gender (*p* = 0.59), age (*p* = 0.19) and general satisfaction with nursing care and the assessment within all major criteria (*p* > 0.05). It was shown that the satisfaction with nursing care within major criterion II “Care and treatment” (*p* = 0.04) and IV “Parental participation” (*p* = 0.03) were evaluated higher by parents with a lower level of education.

No statistical significance was found for the major criteria I “Information” (*p* = 0.11), III “Availability” (*p* = 0.61), V “Professionalism/Professional approach” (*p* = 0.16) and overall satisfaction assessment (*p* = 0.7).

## 4. Discussion

Outstanding quality of medical services, which is expressed by the satisfaction of patients and their families, is a challenge for modern health care systems [1]. The assessment of satisfaction with medical care, including nursing care is recognized as a part of the integrated quality management system, setting the direction of changes in modern healthcare facilities [1,2].

The results of studies conducted in the last decade in various scientific centres using standardized research tools [8,10,15,33,34] or original survey questionnaires [16] confirm the high level of parental satisfaction with nursing care or its specific aspects. The results of our research are in line with the trend of the high level of overall parental satisfaction with nursing care.

For the individual major criteria: I “Information”, II “Care and Treatment”, III “Availability”, IV “Parental participation” and V “Professionalism/Professional approach” the score of assessment ranged from 4.11 to 4.38 points. Similarly, like in the study conducted by Smoleń & Ksykiewicz-Dorota [10], the criterion III “Availability” was the highest evaluated by parents. However, the outcomes of the analysis of individual specific criteria turned out to be lower than expected. The lowest score was given for the specific criterion “The nurse introduced herself by name and surname” within the major criterion V “Professionalism/Professional approach”.

Finding the reasons for these results would require further research in this area. Perhaps the optimization of activities aimed at making the staff aware of the importance to introduce themselves by name and surname will be a factor improving the perception of the quality of nursing care. It is also relevant that the nursing team provide informational and emotional support [16,34] in which they have full competence [35,36]. It is also necessary to cooperate with parents based on their expectations and prepare them to take care of the child after discharge from the hospital, and this was confirmed by other scientific reports [15,37].

In our study, parents of children over 6 years of age gave higher scores for all the criteria evaluating satisfaction with care. Similar results were obtained by Uysal & Cirlak [33] who proved that parents of seven-year-olds and older children showed higher level of satisfaction with nursing care than parents of children under the age of 7 years. Despite different cultural considerations in the examined groups and various standardized research tools applied in the studies the findings were similar in this aspect, which may confirm that children’s age is a universal factor which affects satisfaction from nursing. Therefore, these results can be explained by the specificity of the early developmental periods, which has an impact on the nature of symptoms, adaptation of children to hospital conditions and their reactions to separation from the family environment. These factors may generate higher expectations of parents towards nursing teams [38]. A disparity between expectations of parents and the care they actually receive results in a lower score in the assessment of satisfaction with services. Also, the development of parental caring competence as a child grows up and the process of gaining experience, also related to illness, imply a higher evaluation of services [36]. However, other scientific reports did not confirm the relationship between the age of hospitalised children and parental satisfaction with care [8,34]. This can be caused by an influence of dependent and independent variables, other than those applied in our study, including a significantly smaller number of parents who took part in the study.

Our research proves that parents of children admitted to the hospital in an emergency mode gave lower score for the individual criteria of satisfaction with care (I “Information”, II “Care and Treatment”, IV “Parental participation”) and general satisfaction with care. An emergency admission to the hospital, which is usually associated with a sudden onset of illness or deterioration of the child’s health, does not allow for the physical and mental preparation of parents and children for hospitalisation, hindering the process of adaptation and causing fear, which may have an impact on the satisfaction with nursing services. However, Smoleń & Ksykiewicz-Dorota [10] obtain different outcomes. According to the reports provided by them, parents of children admitted to the hospital in an emergency mode higher assessed the possibility of participating and making decisions regarding care than parents of children admitted in an elective mode.

The study found no correlation between the satisfaction with nursing care and the duration of the child’s stay in the hospital. According to other scientific reports, the evaluation of the relationship between the satisfaction with care and the duration of hospitalisation was different [8,10,26], which may have been the result of other inclusion criteria to the study group, the use of different research tools or the small size of the study group. It should be pointed out that the longer the children stay in hospital, the better they are adapted to hospitalisation conditions, which is also true about their parents/legal guardians who participate in hospital nursing and treatment. However, when the hospitalisation period is prolonged due to complications, hospital infections or other latrogeny factors, both parents’ and patients’ reactions can become negative and determine a lower level of satisfaction with nursing. This aspect should definitely be taken into account in further studies.

Like in the studies conducted by other authors, the age and sex of parents were not determinants of the satisfaction with nursing care [16,33,34,39]. However, there are no unambiguous reports in this area [10,12,40]. The diversity of outcomes in research reports and our study results may be influenced by the choice of a research tool, as well as the family model (patriarchal/matriarchal/partnership) or cultural/national influences.

On the other hand, parental education was an important determinant of the level of satisfaction with nursing care. Parents with lower education were more satisfied with services. These results are consistent with the reports provided by other authors [10,41,42]. Perhaps lower parental education is associated with lower awareness of the child’s and parental rights in the hospital, the lack of knowledge about the developmental specificity, and consequently less expectations about the nursing team. However, according to the scientific reports provided by Uysal & Cirlak [33] and Aslanabadi & Shahbazi [34], parental education was not related to the level of satisfaction with care.

The analysis of scientific reports and our results indicate that parents of hospitalised children have similar expectations regarding nursing care [8,10,15,16], although their scope may be different and dependent not only on the specificity of the disease, but also on its course, the child’s health [26,43], emotional condition of parents [12,44] and previous experiences of hospitalisation [8,36,45]. Moreover, the level of organization of health care in Poland is comparable in all hospitals, which is also true about the health insurance system. Also, there is little diversity in cultural and religious background of the society. While comparing the results of the study with the studies conducted by other authors it should be pointed out that the observed differences may result not only from the choice of research tools used for assessing satisfaction but also from patients’ and their parents’ expectations, which is determined by a general level of medical care provided, organization of the health care system and also cultural factors. However, scientific research proves that hospitals with good work environments and better professional nurse staffing have more satisfied patients and nurses, and evidence of better quality and safety of care, despite differences in how healthcare is organised, financed and resourced, and in despite of cultural differences [46]. In light of the aforementioned findings, further research in this area is recommended.

### 4.1. Clinical Implications

The standardized research tool used in the study is available to nursing teams, it does not require psychologists’ interpretation, is understandable and easy to use for parents. The awareness of determinants of parental satisfaction will allow for objectivization of results and introduction of measures to optimize both patient and family-oriented services.

### 4.2. Limitations

The study results should be treated as preliminary and cannot be used to draw general conclusions on parental satisfaction with nursing due to the following: Limited group of subjects, the lack of respondents from small or provincial paediatric hospitals, no assessment of the subjects’ emotional state, which may have an impact on their expectations towards nursing care and to determine aforementioned satisfaction, the omission of the parents’ earlier experiences of hospitalisations and their influence on the current perception of nursing care.

## 5. Conclusions

The result of the overall assessment of parental satisfaction with nursing care was high. The highest scores were given for the major criterion III “Availability”, while the lowest score was for the major criterion II “Care and Treatment” and IV “Parental participation”. The age of the child, the mode of admission and education of the respondents are significant predictors of parental satisfaction with nursing care.

## Figures and Tables

**Table 1:** Characteristics of the study group.

Sociodemographic Variables of the Subjects		[%]	[*n*]
Gender	woman	84.5	284
	man	15.5	52
Age of parents	up to 30 years of age	30.7	103
	30-40 years	46.4	156
	above 40 years of age	15.5	52
	no data available	7.4	25
Education	higher	86.9	292
	secondary	11.6	39
	vocational	1.5	5
Age of child	neonate	1.5	5
	infant	30.4	102
	toddlers and kindergarten period	42.6	143
	early school period	17.3	58
	puberty	8.3	28
Duration of hospitalisation	3–7 days	59.8	201
	8–14 days	24.4	82
	over 14 days	15.8	53

%—response rate; *n*—number of valid answers.

**Table 2:** Parental satisfaction with nursing team care and the age of hospitalised children.

Child’s Age		Criterion Information	Criterion Care and Treatment	Criterion Availability	Criterion Parental Participation	Criterion Professionalism/Professional Approach	General Satisfaction
Up to 6 years	M	4.04	4.05	4.35	4.05	4.17	4.13
	Me	4.19	4.11	5.00	4.17	4.32	4.26
	*n*	250.00	250.00	250.00	250.00	250.00	250.00
	SD	0.85	0.81	0.80	0.78	0.68	0.69
Over 6 years	M	4.39	4.31	4.45	4.28	4.39	4.36
	Me	4.50	4.56	5.00	4.33	4.53	4.47
	*n*	86.00	86.00	86.00	86.00	86.00	86.00
	SD	0.56	0.71	0.79	0.68	0.56	0.57
Total	M	4.13	4.11	4.38	4.11	4.23	4.19
	Me	4.25	4.28	5.00	4.17	4.37	4.32
	*n*	336.00	336.00	336.00	336.00	336.00	336.00
	SD	0.80	0.79	0.79	0.76	0.66	0.67
*p* U Mann- Whitney		0.002	0.01	0.30	0.01	0.01	0.01

M—arithmetic mean; Me—median; *n*—number of valid answers; SD—standard deviation; Min—minimum value; Max—maximum value; *p*—significance level.

**Table 3:** Parental satisfaction with nursing team care and the mode of admitting a child to the hospital.

Mode of Admitting a Child to the Hospital		Criterion Information	Criterion Care and Treatment	Criterion Availability	Criterion Parental Participation	Criterion Professionalism/Professional approach	General Satisfaction
Elective	M	4.03	4.06	4.35	4.06	4.20	4.14
	Me	4.13	4.22	5.00	4.17	4.37	4.26
	*n*	224.00	224.00	224.00	224.00	224.00	224.00
	SD	0.85	0.81	0.78	0.77	0.68	0.68
Emergency	M	4.33	4.23	4.42	4.22	4.28	4.30
	Me	4.50	4.33	5.00	4.33	4.37	4.40
	*n*	112.00	112.00	112.00	112.00	112.00	112.00
	SD	0.66	0.74	0.82	0.73	0.61	0.63
Total	M	4.13	4.11	4.38	4.11	4.23	4.19
	Me	4.25	4.28	5.00	4.17	4.37	4.32
	*n*	336.00	336.00	336.00	336.00	336.00	336.00
	SD	0.80	0.79	0.79	0.76	0.66	0.67
*p* U Mann- Whitney		0.003	0.05	0.30	0.04	0.34	0.03

M—arithmetic mean; Me—median; *n*—number of valid answers; SD—standard deviation; Min—minimum value; Max—maximum value; *p—*significance level.
